# A multi-country implementation research initiative to jump-start scale-up of outpatient management of possible serious bacterial infections (PSBI) when a referral is not feasible: Summary findings and implications for programs

**DOI:** 10.1371/journal.pone.0269524

**Published:** 2022-06-13

**Authors:** Yasir Bin Nisar, Samira Aboubaker, Shams El Arifeen, Shabina Ariff, Narendra Arora, Shally Awasthi, Adejumoke Idowu Ayede, Abdullah H. Baqui, Ashish Bavdekar, Melkamu Berhane, Temsunaro Rongsen Chandola, Abadi Leul, Salim Sadruddin, Antoinette Tshefu, Robinson Wammanda, Assaye Nigussie, Lee Pyne-Mercier, Luwei Pearson, Neal Brandes, Steve Wall, Shamim A. Qazi, Rajiv Bahl

**Affiliations:** 1 Department of Maternal, Newborn, Child and Adolescent Health and Ageing, World Health Organization (WHO), Geneva, Switzerland; 2 Consultant (Retired WHO Staff), Geneva, Switzerland; 3 International Centre for Diarrhoeal Disease Research Bangladesh (icddr,b), Dhaka, Bangladesh; 4 Pediatrics and Child Health, Aga Khan University, Karachi, Pakistan; 5 The INCLEN Trust International, New Delhi, India; 6 Department of Pediatrics, King George’s Medical University, Lucknow, Uttar Pradesh, India; 7 Department of Paediatrics, College of Medicine, University of Ibadan and University College Hospital, Ibadan, Nigeria; 8 Department of International Health, Bloomberg School of Public Health, Johns Hopkins University, Baltimore, Maryland, United States of America; 9 Vadu Rural Health Program, KEM Hospital Research Centre, Pune, Maharashtra, India; 10 Department of Pediatrics and Child Health, Jimma University, Jimma, Ethiopia; 11 Centre for Health Research and Development, Society for Applied Studies, Kalu Sarai, New Delhi, India; 12 Department of Paediatrics and Child Health, School of Medicine, Mekelle University, Mekelle, Ethiopia; 13 Retired WHO Staff, Toronto, Canada; 14 Department of Community Health, Kinshasa School of Public Health, Kinshasa, DR Congo; 15 Department of Community Medicine, Ahmadu Bello University Teaching Hospital, Ahmadu Bello University, Zaria, Nigeria; 16 Health science college, Bahir Dar University, Bahir Dar, Ethiopia and Harvard, T.H. CHAN School of Public Health; Boston, Massachusetts, United States of America; 17 Bill and Melinda Gates Foundation, Seattle, Washington, United States of America; 18 UNICEF, HQ, New York, New York, United States of America; 19 USAID, Washington, DC, United States of America; 20 Save the Children, Saving Newborn Lives, Washington, DC, United States of America; SRM Institute of Science and Technology, INDIA

## Abstract

**Introduction:**

Research on simplified antibiotic regimens for outpatient treatment of ‘Possible Serious Bacterial Infection’ (PSBI) and the subsequent World Health Organization (WHO) guidelines provide an opportunity to increase treatment coverage. This multi-country implementation research initiative aimed to learn how to implement the WHO guideline in diverse contexts. These experiences have been individually published; this overview paper provides a summary of results and lessons learned across sites.

**Methods summary:**

A common mixed qualitative and quantitative methods protocol for implementation research was used in eleven sites in the Democratic Republic of Congo (Equateur province), Ethiopia (Tigray and Oromia regions), India (Haryana, Himachal Pradesh, Maharashtra, and Uttar Pradesh states), Malawi (Central Region), Nigeria (Kaduna and Oyo states), and Pakistan (Sindh province). Key steps in implementation research were: i) policy dialogue with the national government and key stakeholders, ii) the establishment of a ‘Technical Support Unit’ with the research team and district level managers, and iii) development of an implementation strategy and its refinement using an iterative process of implementation, programme learning and evaluation.

**Results summary:**

All sites successfully developed and evaluated an implementation strategy to increase coverage of PSBI treatment. During the study period, a total of 6677 young infants from the study catchment area were identified and treated at health facilities in the study area as inpatients or outpatients among 88179 live births identified. The estimated coverage of PSBI treatment was 75.7% (95% CI 74.8% to 78.6%), assuming a 10% incidence of PSBI among all live births. The treatment coverage was variable, ranging from 53.3% in Lucknow, India to 97.3% in Ibadan, Nigeria. The coverage of inpatient treatment ranged from 1.9% in Zaria, Nigeria, to 33.9% in Tigray, Ethiopia. The outpatient treatment coverage ranged from 30.6% in Pune, India, to 93.6% in Zaria, Nigeria. Overall, the case fatality rate (CFR) was 14.6% (95% CI 11.5% to 18.2%) for 0-59-day old infants with critical illness, 1.9% (95% CI 1.5% to 2.4%) for 0-59-day old infants with clinical severe infection and 0.1% for fast breathing in 7–59 days old. Among infants treated as outpatients, CFR was 13.7% (95% CI 8.7% to 20.2%) for 0-59-day old infants with critical illness, 0.9% (95% CI 0.6% to 1.2%) for 0-59-day old infants with clinical severe infection, and 0.1% for infants 7–59 days old with fast breathing.

**Conclusion:**

Important lessons on how to conduct each step of implementation research, and the challenges and facilitators for implementation of PSBI management guideline in routine health systems are summarised and discussed. These lessons will be used to introduce and scale-up implementation in relevant Low- and middle-income countries.

## Introduction

Severe infections including pneumonia, sepsis and meningitis in neonates caused around 500,000 deaths in 2019 [1]. An estimated 6.9 million episodes of clinically suspected severe infection, called Possible serious bacterial infection (PSBI) by the World Health Organization (WHO), occurred in 2012 [2]. The WHO Integrated Management of Childhood Illness (IMCI) 2014 protocol recommends that young infants 0–59 days of age who have PSBI should be referred for inpatient treatment with injectable antibiotics for at least 7–10 days and other supportive care [3,4].

In many low resource settings, inpatient care is neither feasible nor acceptable for many families [5–11], resulting in low coverage of treatment for PSBI. Based on results of several trials in Africa and Asia showing that outpatient treatment is safe and effective for most cases of PSBI when a referral is not feasible [7–11], WHO published guidelines for outpatient PSBI treatment in such a situation in 2015 [12]. The young infant component of the IMCI chart booklet was accordingly revised [13].

There was a need for implementation research to understand the implications of adopting and implementing the PSBI outpatient management guideline in programme settings. WHO therefore led a multi-organisation implementation research initiative in seven countries, supported by Bill & Melinda Gates Foundation, Saving Newborn Lives/Save the Children USA, Global Affairs Canada, and USAID. The research was conducted by local research institutions, in close partnership with national and local policymakers, programme managers and implementers and has been published (or under review [14]) as a collection in PLOS One [15–23] and elsewhere [24]. This paper provides summary results, lessons learned and program implications from all sites.

## Methods summary

All study sites received ethical approval from local ethics committees. Additional ethical approval was obtained from the WHO ethics review committee. Written informed consent was obtained from all eligible caregivers prior to their participation in the study. To ensure confidentiality and anonymity during analysis, no personal identifiers were entered into the study database and only study personnel had access to the data.

Implementation research studies were carried out from October 2015 to March 2019 in 11 sites in six African and Asian countries, i.e., in Bominenge and Karawa Health Zones in North and South Ubangi (Equateur province) in DR Congo [14], Jimma (Oromia Region) [15] and Mekelle (Tigray Region) [16] in Ethiopia, Palwal (Haryana) [17], Sirmaur (Himachal Pradesh) [18], Pune (Maharashtra) [19] and Lucknow (Uttar Pradesh) [20] in India, Ntechu (Central Region), Malawi [21], and Zaria (Kaduna) [22] and Ibadan (Oyo) [23] in Nigeria and Thatta (Sindh), Pakistan [24]. Each study site had a population of 100,000 to 300,000, with a combined population of over 2.5 million.

This research aimed to develop an implementation strategy for WHO guidelines on inpatient and outpatient treatment of PSBI to improve treatment coverage, which could be subsequently scaled up. The programmatic targets of this strategy were that (i) health facilities will provide inpatient or outpatient management of young infants with signs of PSBI, (ii) at least 80% of the estimated number of young infants with PSBI will receive treatment, and iii) at least 80% of the young infants with PSBI who are treated will receive adequate quality treatment. Appropriate treatment was defined as the provision of inpatient treatment (injectable gentamicin and ampicillin or any other appropriate antibiotics as advised by the treating physician or according to the hospital policy) or outpatient treatment (IM gentamicin injection for at least two days and oral amoxicillin twice daily for at least five days plus) to sick young infants with PSBI when the referral was not feasible.

The implementation strategy was developed based on the conceptual framework adapted from the Research Effectiveness Adoption Implementation Maintenance RE-AIM framework [25]. The WHO-UNICEF operationalisation guide for PSBI treatment provided a systematic approach to the introduction and early steps in learning, quality control, integration of services, and monitoring/supervision [26].

Policy dialogue with central, state/province/region and district health authorities was held in all countries before initiation of implementation research to obtain approval and consensus for outpatient treatment of PSBI when a referral is not feasible. At these policy dialogue meetings, findings of relevant research, WHO guidelines for managing PSBI in young infants when a referral is not feasible and experience of implementing these guidelines in other countries was shared with the national Ministry of Health, other stakeholders like professional paediatric and neonatal associations by experts from WHO and research teams.

A baseline assessment of the barriers to the implementation of guidelines for inpatient and outpatient treatment of PSBI intervention was conducted at all sites. This assessment used a mix of qualitative and quantitative methods to understand the knowledge, attitude and practices of the families regarding illnesses in young infants and health care seeking, and available health facility infrastructure, commodities, services, and practices for management of sick young infants (Panel 1). The findings of the baseline assessment were used in the development of the implementation strategy.

**Panel 1. Preparation for and implementation of outpatient management of young infants with signs of PSBI in programme settings.**

- Selection of early implementation sites by the relevant governments.
- Selection of academics/research teams by the government to provide technical support to the implementors.
- Adoption and integration of the World Health Organization (WHO) Possible Serious Bacterial Infection (PSBI) guideline into the national or regional integrated management of childhood illness (IMCI) tools.
- Establishment of Technical Support Units and teams.
- Orientation and dialogue with national and sub-national implementers, health workers and the community.
- Baseline surveys and situation analysis of the readiness of the health system.
- Strengthen the surveillance of pregnant women and births where needed.
- Where required, institute or support home visits by community health workers (CHW), identification of sick young infants and their referral to the nearest health facility.
- Empower caregivers to identify their sick young infants through education and counselling to seek timely appropriate care-seeking.
- Strengthen linkage of the first level health facilities to the referral level hospital.
- Prepare the first-level health/primary health care facilities to provide outpatient treatment of PSBI near patients’ homes when a referral to the hospital was not feasible.
- Orient and build the capacity of the nurses, physicians, other relevant health workers, including CHWs (where relevant) and supervisors to support the delivery of the interventions.
- Acquisition of necessary commodities and medicines, storage, record keeping and replenishment.
- Identify mechanism for follow-up, supervision and monitoring of activities.
- Preparation for mentoring and clinical skills retention of the health workers providing the outpatient treatment.
- Establishment of feedback and problem-solving mechanisms.

The intervention was implemented in routine program settings. A ‘Technical Support Unit’ (TSU), including the state and district health officials and researchers, was established and provided inputs on strategy, technical oversight and support for this implementation research (Panel 2). The government health services implemented the guideline, and the research team provided support through regular inputs on the fidelity of the implementation and problem identification. Solutions to identified problems were developed in close collaboration with the government.

**Panel 2. Role of the Technical Support Unit (TSU) in the implementation research.**

- Baseline surveys and situation analysis to assess the readiness of the health system to implement the new World Health Organization (WHO) Possible Serious Bacterial Infection (PSBI) management guideline [12].
- Assist the government in establishing human resource requirements.
- Develop a training programme for capacity building of health workers and by training master trainers in light of recommended WHO PSBI management guidelines.
- Develop tools and job aids for the health workers to support the implementation of WHO PSBI management guidelines.
- Identify needs and requirements for the implementation of the guideline.
- Support the implementation of the guideline.
- Provide logistic support and commodities related to the outpatient management of young infants with PSBI, where required.
- Develop supervisory tools and training of supervisors.
- Provide technical support in identifying gaps in the clinical skills of the health workers and provide mentoring and hand-holding for reinforcing their skills.
- Develop indicators related to PSBI management and support in their measurement.
- Establish a programme learning platform through monitoring the quality of implementation, documenting the progress and identifying gaps and challenges.
- Assist the government in problem-solving by finding solutions and refining the implementation strategy where needed.
- Collect qualitative and quantitative data, conduct data management and analysis to document implementation research and clinical outcomes.
- Disseminate the key findings and programme learnings for potential scale-up of the new guideline at the local, regional and national levels.

The TSU performed the following three major functions at all study sites. Only in Himachal Pradesh, India, these functions were performed by mutually exclusive teams i.e., implementation support team, program learning team and monitoring and evaluation team. The implementation support was provided through supporting the health system to implement the PSBI intervention by facilitating the training and community awareness activities and checking the availability of supplies and commodities (Panel 3). The program learning function was performed through conducting in-depth interviews with the families, community health workers (CHW), health workers, and program managers made direct observations to assess the quality of implementation. The monitoring and evaluation function independently captured information of live births in the study population, infants identified to have PSBI and their treatment from the health facility record and outcome of the illness through interviews with the families.

**Panel 3. Implementation of outpatient management of young infants with signs of PSBI in programme settings.**

- Identification of pregnant women and live births in most sites.
- Identification of sick young infants in the communities and care-seeking either directly by families or through CHWs from the selected first-level health facilities.
- Assessment and classification of sick young infants at selected first-level health facilities.
- Referral to a hospital of sick young infants with PSBI signs who needed a referral.
- Outpatient treatment was provided to those whose parents refused a hospital referral.
- Following up on young infants who received outpatient treatment and documenting outcomes.
- Supervision, hand-holding and mentoring of health workers in delivering the intervention.
- Refresher training of the health workers for management of sick young infants as required.
- Ensuring a regular supply of commodities.
- Programme learning through monitoring and evaluation.
- Problem identification and problem-solving.
- Data collection and analysis.
- Documentation.

## Results summary

### Implementation strategies

All sites successfully developed and evaluated an implementation strategy to increase coverage of PSBI treatment. The common elements of these implementation strategies across sites included: (i) improving recognition of danger signs by CHWs at postnatal home visits, (ii) empowering mothers and families to recognise and seek health care for young infants with danger signs, (iii) building capacity of health workers at first-level health facilities to identify and refer infants with PSBI and build their confidence in treating when a referral is not possible through regular technical support (iv) ensuring uninterrupted availability of devices, medicines and supplies required to identify and treat PSBI, and (v) monitoring quality of PSBI treatment provided.

### PSBI treatment coverage

During the study period, a total of 88179 live births were identified by the study monitoring and evaluation team in the study catchment area of the 11 sites in 6 countries. Assuming a 10% incidence of PSBI among live births during 0–59 days of life, we estimated that about 8818 cases of PSBI occurred in the study populations. The study monitoring and evaluation team documented 6677 young infants who were identified and treated at health facilities in the study area as inpatients or outpatients, leading to a treatment coverage of 75.7% (95% CI 74.8% to 78.6%).

**Fig 1:**
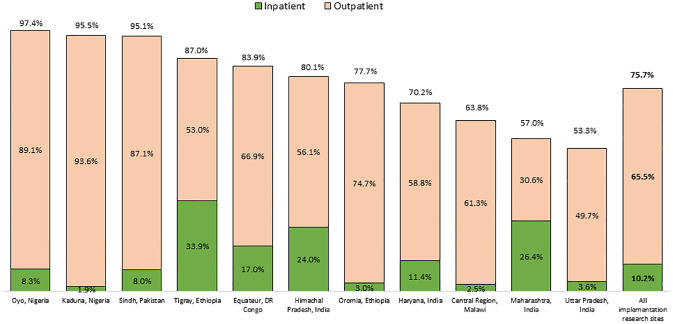
Estimated coverage of possible serious bacterial infection (PSBI) treatment by site, overall and as inpatients or outpatients.

Fig 1 shows that the treatment coverage was variable, ranging from 53.3% in Uttar Pradesh, India [20], to 97.3% in Oyo, Nigeria [23]. The figure also shows the proportion of live births who received PSBI as inpatients or outpatients. The coverage of inpatient treatment ranged from 1.9% in Kaduna, Nigeria [22], to 33.9% in Tigray, Ethiopia [16]. The coverage of outpatient treatment ranged from 30.6% in Maharashtra, India [19], to 93.6% in Kaduna, Nigeria [22].

### Acceptance of referral for inpatient treatment

Of the 7972 young infants identified with signs of PSBI (whether living inside or outside the study catchment area), 7484 young infants were sub-classified (333 infants at Tigray, Ethiopia [16] and 155 at Haryana, India [17] were not sub-classified into critical illness or clinical severe infection). There were 2898 (38.7%) 7–59 days old infants with only fast breathing, 4037 (53.9%) 0–59 days old infants with signs of clinical severe infection (including 988, 0–6 days old infants with only fast breathing) and 549 (7.3%) 0–59 days old infants with signs of critical illness.

**Fig 2:**
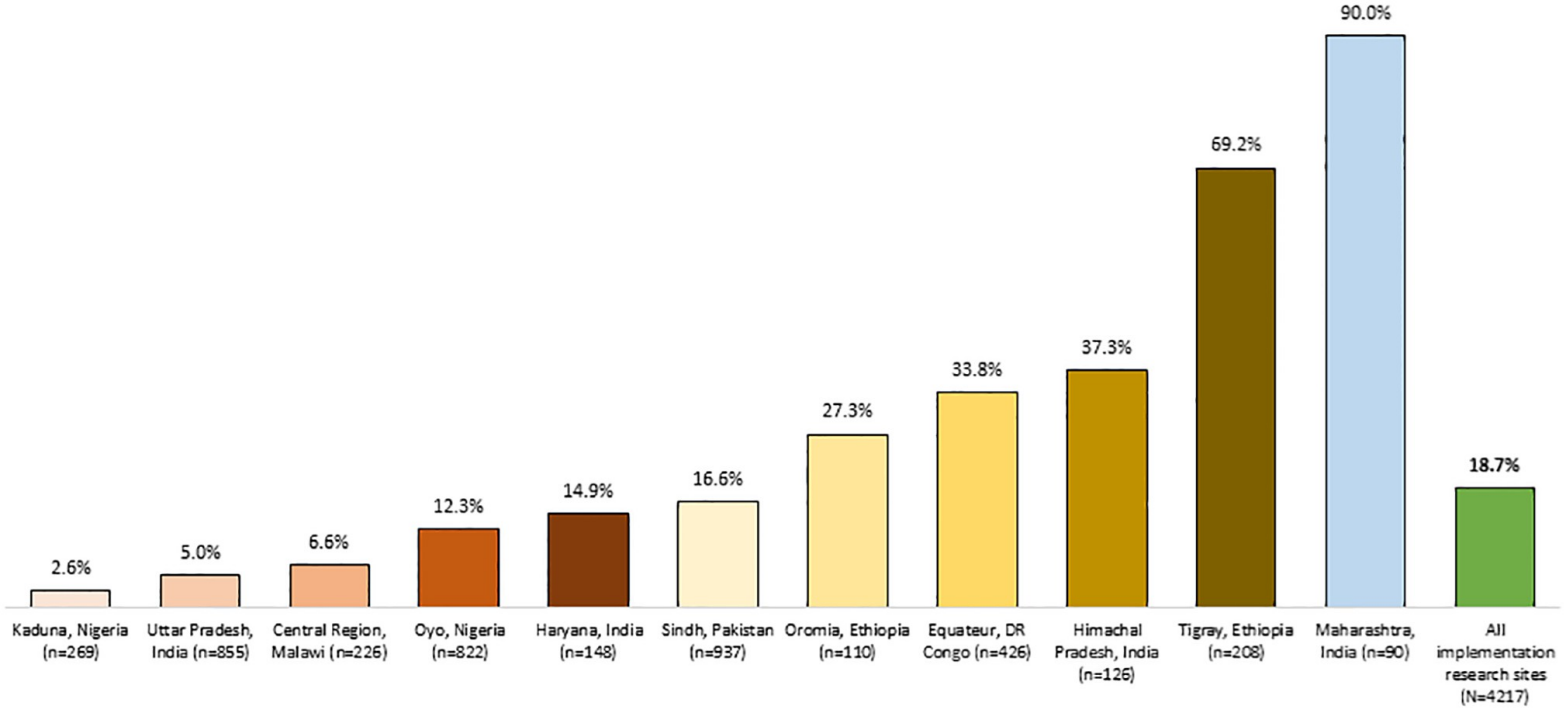
Acceptance of hospital referral among young infants with PSBI, by site.

Of the 7972 infants identified to have PSBI, 4217 (infants with signs of critical illness or clinical severe infection) were referred for inpatient care from a first-level health facility, but the overall referral acceptance rate was 18.7% (95% CI, 17.6% to 19.9%) (Fig 2). The referral acceptance rate was variable across sites, ranging from 2.6% in Kaduna, Nigeria [22], to 90% in Maharashtra, India [19]. Most sites (9 of 11) had a referral acceptance rate of less than 40%. When the referral was not accepted, the only possibility for infants with PSBI to receive treatment was simplified antibiotic regimens as outpatients.

### The outcome of infants identified to have PSBI

The vital status of all infants with PSBI was assessed two weeks after their identification, including those who received inpatient or outpatient treatment. Overall, the case fatality rate (CFR) for all cases of PSBI was 2.2% (95% CI 1.9% to 2.6%). 171 deaths were reported from 11 sites among 7685 cases of PSBI for whom outcome assessment data was available. The CFR was 14.6% (95% CI 11.5% to 18.2%) for 0-59-day old infants with critical illness (452 cases and 66 deaths), and 1.9% (95% CI 1.5% to 2.4%) for 0-59-day old infants with clinical severe infection (3904 cases and 75 deaths). Only one study from India [18] reported two deaths among 7–59 days old infants with only fast breathing leading to CFR of 0.1% (0.01% to 0.2%), (2841 cases and 2 deaths). Among those who were not sub-classified to critical illness or clinical severe infection and received inpatient treatment, thus not included in CFR by sub-group, there were 28 deaths among 333 infants in Tigray, Ethiopia [16], and no death among 155 infants in Haryana, India [17].

#### The outcome of infants with PSBI treated as inpatients

Among infants treated as inpatients (n = 879) who were sub-classified, and their outcome was assessed, the CFR was 15.0% (95% CI 11.2% to 19.6%) for 0-59-day old infants with critical illness (299 cases and 45 deaths), 8.0% (95% CI 6.0% to 10.6%) for 0-59-day old infants with clinical severe infection (569 cases and 46 deaths), and no death was reported among 7–59 days old infants with only fast breathing across all sites (only 11 cases received inpatient treatment).

#### Outcomes of young infants with signs of PSBI treated on an outpatient basis

Among infants treated as outpatients (n = 6318) who were sub-classified, and the outcome was assessed, the CFR was 13.7% (95% CI 8.7% to 20.2%) for 0-59-day old infants with a critical illness (153 cases and 21 deaths), and 0.9% (95% CI 0.6% to 1.2%) for 0-59-day old infants with clinical severe infection (3335 cases and 29 deaths) across all sites. Only one study from India [18] reported two deaths, 0.1% (95% CI 0.01% to 0.2%) among 7–59 days old infants with only fast breathing (2830 cases and 2 deaths).

Additionally, non-fatal clinical treatment failure (defined as deterioration within the first seven days, or persistence of signs of PSBI on day 8, after initiation of treatment) was assessed in infants who received outpatient PSBI treatment (n = 6318). Non-fatal clinical treatment failure rates were 11.1% (95% CI 6.6% to 17.2%) for 0–59 days old with critical illness (153 cases and 17 treatment failures), and 5.2% (95% CI 4.5% to 6.0%) for clinical severe infection (3335 cases and 173 treatment failures). A substantial proportion of 7–59 days old sick young infants presenting at the first level health facilities had only fast breathing, which was treated with oral amoxicillin without referral advice (n = 2830). Nearly all responded well to the oral amoxicillin and improved, and the overall treatment failure rate (including deaths) was 3.2% (95% CI 2.6% to 3.9%). Death was only reported in two cases seen in Himachal Pradesh, India [18]. This supports the WHO recommendation that these young infants can be treated with oral amoxicillin on an outpatient basis without being referred to a hospital [12,13].

## Discussion

### Main findings and their impact

High coverage of PSBI treatment was achieved at all sites when an implementation strategy was developed and implemented to identify young infants with signs of PSBI and refer them for inpatient treatment and provide outpatient treatment when a referral was not feasible. Acceptance of referral continues to be difficult in these sites, and therefore outpatient treatment contributed substantially more than inpatient treatment to coverage. Outpatient treatment was associated with relatively low mortality and non-fatal treatment failure.

Implementation research results were disseminated at two regional workshops (Addis Ababa, New Delhi) as well as at national meetings in all the countries where this research was implemented. In the workshops, a scale-up plan to implement outpatient management of PSBI when a referral was not feasible was developed as part of the national plan for maternal, newborn and child health programmes. Bangladesh, DR Congo, Malawi, Nigeria and Pakistan adopted PSBI management when a referral was not feasible in their national policy.

### Possible reasons for variation in coverage and referral acceptance

Three sites, two in Nigeria and one in Pakistan showed overall treatment coverage of over 95%, mostly due to high outpatient treatment (87.1–93.6%) and low hospital treatment (1.9%-8.3%) [22–24]. In India, Pune and Lucknow [19,20], the coverage of treatment was 57% and 54%, respectively, most probably because of lower than expected identification of PSBI cases, especially the fast breathing pneumonia cases, compared to the other sites.

Six sites reporting referral acceptance rates of <20% [17,20–24] were similar to those reported earlier from several African and Asian countries [5,6,8–11]. The highest referral acceptance rate in Pune, India, was due to an effective referral system, good communication between the care providers and families and awareness among families about the seriousness of illness [19]. Whereas in Tigray, Ethiopia, free ambulance service, active women development groups and free treatment for newborns were cited as the reasons for high referral acceptance [16]. Referral acceptance in the Himachal Pradesh, India [18] and DR Congo [14] sites was mostly due to a higher proportion of critically ill infants.

### Comparison of outcome of infants with PSBI with previous studies

The overall death rate was 2.1% (95% CI 1.3% to 2.9%), which was substantially less than 9.8% reported in a systematic review of PSBI from low and middle-income countries (LMICs) in Africa, Asia and Latin America [2]. We believe that the potential reasons for this low death rate were: early recognition of young infants with danger signs by the families or CHWs, prompt care-seeking from trained healthcare providers and timely and appropriate treatment either at a hospital or at health facilities near to patient’s home when a referral to a hospital was not feasible. Among those young infants who were treated on an outpatient basis, the clinical treatment failure varied between sites, but it was less than 10% in most of them. Four sites didn’t report any death. Although the highest treatment failure rate of 22.2% was reported from Pune, India [19], it was due to two deaths out of nine cases who received outpatient treatment.

### Cost of PSBI treatment

In the current PLoS One Neonatal Infection Collection, Garg and colleagues [27] estimated the costs of treating young infants with PSBI as outpatients when a referral is not possible in low-resource settings using the data from African Neonatal Sepsis Trials (AFRINEST) [10,11]. The weighted average costs across all sites for treating clinical severe infection in 2012 were US$ 25.3 (95% CI US$ 20.4–30.1) per infant treated when the regimen with 7 days of gentamicin plus amoxicillin was used, and US$ 20.9 (95% CI US$16.4–25.3) when the regimen with 2 days gentamicin plus 7 days amoxicillin was used. The weighted average cost for treatment of 7-59-day old infants with fast breathing with oral amoxicillin was US$ 18.3 (95% CI US$ 13.4–23.3). The corresponding costs per treated young infant in 2018, when adjusted for inflation, were US$ 39.3, US$ 32.5, and US$ 29.0, respectively.

### Lessons related to conducting implementation research

Several key lessons were learned from the process of initiating, designing, implementing and coordinating this multi-partner implementation research, which would be useful for the countries interested in either adopting the PSBI management guideline or scaling up its implementation.

#### Policy dialogue

The policy dialogue was essential for introducing the WHO guideline on PSBI treatment in countries. It was important that in addition to decision-makers and programme managers, the scientific community, professional associations and donors participated, reviewed the evidence, debated on policy options, made and owned decisions for initial implementation. The concern about antimicrobial resistance was often discussed and debated. All countries welcomed the opportunity to obtain local data and implementation experience. The usual result of this policy dialogue was national guidelines for the implementation of PSBI treatment strategy for the implementation districts. At times, the result was a change of national guidelines, as was the case in India, where an addendum to the national guidelines of 2014 was issued in 2017 after an extensive policy dialogue [28,29]. After completion of the implementation research in Ethiopia, the Government of Ethiopia revised their national guideline [30], to only oral amoxicillin for the treatment of fast breathing pneumonia, for which the previous recommendionwas treatment with injectable gentamicin and injectable ampicillin. Similarly, Bangladesh [31,32], Pakistan [24], Nigeria [22,23] and Malawi [21] also changed their national guidelines after the implementation research.

#### Preparatory phase

Baseline assessment of barriers and facilitators for implementation was critical for the design of an effective implementation strategy. The establishment of a partnership between implementers and the scientific and technical community (the Technical Support Unit) was an important and innovative strategy for the implementation of a guideline that challenges the norm that PSBI can only be treated in a hospital. The introduction of this guideline required confidence, technical skills and hand-holding of health workers.

#### Implementation phase

Across all sites, existing health facilities and staff of public health systems were used, without creating a parallel implementation system for the study. The TSU trained health workers on the management of infants with PSBI, built the implementation strategy with programme implementers, actively engaged in identifying gaps and problem-solving, thereby building the confidence of health care providers and programme managers. This partnership provided an opportunity for learning by doing, facilitating the engagement of a wide group of stakeholders and building confidence for implementation. Strategies to monitor early performance and targeted support, particularly the underperforming facilities, was critical when introducing the relatively complex guideline in new settings.

The TSU played an active role in strengthening monitoring, supervision and quality assurance, and problem-solving. Mentoring of health workers by the TSU and hand-holding built their confidence in improving the treatment of sick young infants, including the delivery of injections and follow up. The TSU had to play a greater role in implementation when the health system was very weak [14,22,23], which led to high treatment coverage in this implementation research, but this solution is not sustainable during scale-up.

Community sensitisation was an essential component of implementation. Across all sites, a dialogue with the community was organised to orient the community on the availability of services for sick infants and to promote care-seeking from an appropriate provider. Sometimes, communities participated in the provision of health services. For example, in Nigeria, the community was able to provide a place for a health post where the health workers provided services [22]. In Malawi, the secret mothers’ group supported CHWs in identifying births [21]. Caregiver perception of trust and health workers’ inter-personal communication skills were influential in caregiver acceptability of treatment advice. Community ownership and engagement provided opportunities for problem-solving and the acceptability of the intervention.

Home visits for postnatal care by adequately trained CHWs were important in identifying sick young infants and referring them to the appropriate health care facilities for prompt and timely care. They were also important for empowering mothers and family members through counselling and teaching them about the PSBI signs and where and when to seek care. Despite these efforts, the identification of young infants with fast breathing was substantially lower than expected in a few sites [17–20].

When implementation was well-established in study sites, they served as learning sites for district health managers and health workers from other geographical areas in the country [22,23,33]. Some specific lessons learnt from similar implementation research conducted in several districts in Bangladesh, which was not part of the WHO coordinated work, are given in Panel 4.

**Panel 4. Lessons from implementation research in Bangladesh.**

Lessons from four implementation research studies conducted in Bangladesh are summarized below. Rahman et al. reported on various steps that were needed to implement quality outpatient PSBI management per national guidelines at a district level in Kushtia, Bangladesh, through implementation research [31]. Care-seeking from private providers, predominantly village health doctors, was high. Facility readiness, including health care provider knowledge and skills, was strengthened, but the authors identified that the health care providers did not consistently adhere to the guidelines. They recommended the need to improve private sector management of PSBI cases and improve linkages between private and public sector providers.

Applegate et al. described the programme’s feasibility and acceptability through an analysis of service delivery data [34]. As the programme matured, with improved awareness and acceptability of services among families there was a trend for an increase in utilisation. Uncertainty around treatment outcomes especially for referred cases was identified as a major barrier to monitoring the long-term effectiveness of the programme. Authors recommended that systems were needed for routinely tracking sick young infants that accepted referrals to ensure that families reached the referral facilities and received appropriate care. It was also essential to understand the complexities around referral feasibility and acceptability of the simplified treatment.

Applegate et al. reported on the assessment of caregiver acceptability of the national PSBI guidelines in three rural sub-districts of Bangladesh during early implementation [35]. Caregivers accepted and were satisfied with the simplified antibiotic treatment when a referral was not feasible. Trust in health care providers and inter-personal communication were influential in caregiver acceptability of care. Referral acceptance was not common. Some health care providers engaged informal providers in providing the second-day injectable antibiotic to promote treatment adherence. Mandatory follow-up on day 4 of treatment was reported in two-thirds of the sick young infants.

In another paper, Applegate et al. reported the facility readiness and provider performance in three rural sub-districts of Bangladesh [36]. At baseline, none of the facilities had an adequate supply of antibiotics, which improved with time. Classification and dosage errors were common in the beginning and decreased over time. Some providers had concerns about the efficacy of simplified antibiotic regimens. The authors recommended monitoring early performance and provision of technical targeted support to enhance implementation fidelity when a complex guideline like PSBI is introduced in new settings.

### Implementation challenges

A variety of challenges, mostly related to the functionality of the health system, were faced during the implementation research; some were site-specific, and some were observed across sites. These are summarized as follows. a) Human resources were either not adequate or demotivated [33]. For instance, health workers in Nigeria did not receive their salaries regularly [22,23]. In DR Congo [14], Malawi [21] and Nigeria [22,23], health workers had to be given monetary incentives by the study to carry out their tasks. b) Interruption in the supply of medicines was a common feature in several countries. In DR Congo [14] and Nigeria [22,23], research funds had to be used to purchase antibiotics, and some gaps had to be filled in one site in India [20]. c) The acceptance of the referral of critically ill infants remained a major challenge in almost all countries, and some critically ill young infants who should have received care in a hospital had to be treated on an outpatient basis [15,17–24]. The acceptance of referral varied with the infection severity. Cost of transport, cost of inpatient care, family decisions, mother’s autonomy and household responsibility, lack of understanding about the severity of disease, poor transportation between the community and the hospital, perceived quality of care at the hospital, the attitude of the health workers, organisational factors (inconsistent availability of providers, long waits, stock-outs) are some of the factors that prevent people from accepting referral to a hospital [35,37–40]. d) In South East Asia, the private sector and informal providers play an important role in delivering services and are often preferred over qualified providers, which was observed in several places [17,19,20,31,36], sometimes resulting in serious adverse outcomes [20]. e) Closure of facilities during weekends and public holidays disrupted the course of treatment, especially when the sick young infants had to receive an antibiotic injection [31,35]. Two sites reported arrangements to provide injections during weekends and holidays [14,16].

### Facilitating factors

We observed several factors that facilitated the research and implementation of the guideline. a) The local scientific community of academics, researchers, and professional associations helped programme implementers contextualise and implement the global guideline through learning by doing. b) The political will and commitment of decision-makers to consider an innovative approach to reducing newborn and infant mortality were conducive to the successful implementation of this intervention [33]. c) The presence and use of the existing strategies for promoting care at the community level, such as postnatal home visits, helped identify sick young infants in time. d) The integration of PSBI management into existing management strategies such as IMCI [28–30,33] and Caring for the Newborn at home was considered extremely important by programme implementers. e) As PSBI guidelines included readily available and affordable medicines and other supplies, this made implementation easier. f) Support from the WHO at all stages of implementation, i.e., from policy dialogue, development of an implementation strategy, capacity building, quality assurance and evaluation, was very valuable and was appreciated by both the government officials and researchers/academics.

### Potential advantages of outpatient treatment with simplified antibiotic treatment

Outpatient treatment has advantages for both patients’ families and the health system when a referral to a hospital is not feasible. Treatment near patients’ homes is quite convenient for families, resulting in lower transport costs, money saved by not staying in the hospital or not buying expensive medicines out of pocket, less likelihood of loss of wages, reduced disruption to family life, especially if there are other children or family members who need care at home [35,37–40]. The health system will have a reduced load as issues with quality of care at the hospitals, limited hospital beds, overburdened staff, non-functional equipment, regular supply of commodities, and patient satisfaction are common challenges [41,42], especially in low resource settings. The cost of outpatient treatment is much less than inpatient treatment [27,43]. Moreover, there is less risk of nosocomial infections with outpatient treatment [44,45].

### Way forward

The lessons learnt from implementation research and estimates of costs of implementation can be used to introduce and scale-up inpatient and outpatient PSBI management as part of the IMCI programme in other geographies and countries to increase access to treatment and reduce neonatal and infant mortality. This has happened in over 20 LMICs so far but scale up efforts need to be accelerated to expand to other countries and achieve maximal impact. Attention to maintenance of quality in service delivery when implementing at scale, and sustainability of such implementation, is crucial.
